# Vascular calcification: pathophysiology and clinical implications

**DOI:** 10.1590/S1679-45082013000300021

**Published:** 2013

**Authors:** Marcel Liberman, Antonio Eduardo Pereira Pesaro, Luciana Simão Carmo, Carlos Vicente Serrano

**Affiliations:** 1 Hospital Israelita Albert Einstein, São Paulo, SP, Brazil; 2 Instituto do Coração, Hospital das Clínicas, Faculdade de Medicina, Universidade de São Paulo, São Paulo, SP, Brazil

**Keywords:** Vascular calcification, Atherosclerosis, Myocardial ischemia, Vitamin D, Renal insufficiency

## Abstract

Vascular calcification in coronary artery disease is gaining importance, both in scientific research and in clinical and imaging applications. The calcified plaque is considered the most relevant form of atherosclerosis within the coronary artery tree and is frequently a challenge for percutaneous intervention. Recent studies showed that plaque calcification is dynamic and is strictly related to the degree of vascular inflammation. Several inflammatory factors produced during the different phases of atherosclerosis induce the expression and activation of osteoblastic cells located within the arterial wall, which, in turn, promote the deposit of calcium. The vascular smooth muscle cells have an extraordinary capacity to undergo osteoblastic phenotypical differentiation. There is no doubt that the role of these factors, as well as the elements of genomics and proteomics, could be a vital strategic point in prevention and treatment. Within this context, we conducted an updating review on coronary calcification focused on pathophysiology, experimental models, and clinical implications of vascular calcification.

## RESUMO

A calcificação vascular na doença arterial coronária está ganhando importância, tanto em pesquisas científicas como em aplicações clínicas e de imagem. A placa calcificada é considerada a forma mais relevante de aterosclerose dentro da árvore arterial coronária e frequentemente apresenta um desafio para a intervenção percutânea. Estudos recentes têm demonstrado que a calcificação da placa é dinâmica e está estreitamente ligada ao grau de inflamação vascular. Vários fatores inflamatórios, produzidos durante as diferentes fases da aterosclerose, induzem a expressão e ativação de células osteoblásticas localizadas na parede arterial, que, por sua vez, promovem a deposição de cálcio. As células do músculo liso vascular possuem uma capacidade extraordinária de sofrer diferenciação fenotípica osteoblástica. Não há dúvida de que o papel desses fatores, bem como os elementos de genômica e proteômica, poderia ser um ponto estratégico fundamental na prevenção e no tratamento. Neste contexto, realizamos uma atualização sobre a calcificação coronária, com foco em fisiopatologia, modelos experimentais e implicações clínicas da calcificação vascular.

## INTRODUCTION

Vascular calcification is a relevant pathophysiological process that is associated with coronary atherosclerosis, and is a prognostic marker of cardiovascular morbidity and mortality^(1–3)^. Calcification determines significant pathophysiological modulation of vascular aging^(4)^, such as, for example, decreased vascular complacency and elevated pulse pressure (by increasing the systolic arterial pressure and decreasing the diastolic pressure), in addition to flow distribution change and loss of other regulation mechanisms.

The objective of this review was to assess the pathophysiological aspects of vascular calcification by presenting prevalent clinical, laboratory and epidemiological conditions, and therefore discuss the main cellular mechanisms and signaling mediators that participate in this process.

## EPIDEMIOLOGICAL RELEVANCE AND ASSOCIATION WITH CARDIOVASCULAR MORTALITY

Vascular calcification, previously considered a passive degenerative condition of aging (Monckeberg 1904), is currently characterized as an active biomineralization process^(5)^, with complex signaling pathways^(6)^, similar to osteogenesis^(7)^. On the epidemiological scenario, vascular calcification increases with age, atherosclerosis, renal failure^(8)^, diabetes mellitus, hypercholesterolemia, osteoporosis, obesity, smoking, menopause, and lack of physical exercise^(9)^. Calcification of the aorta may affect 65% of people in a general population with a mean age of 60 years and correlates with coronary calcification identified by multidetector computed tomography, with a positive predictive value to increase cardiovascular morbidity and mortality in asymptomatic patients at intermediate risk^(2)^. Additionally, calcification of the abdominal aorta is associated with increased cardiovascular mortality, even when adjusted for age^(2)^. Conversely, calcification of the coronary arteries is associated with a greater risk of myocardial infarction and with increased incidence of adverse events during percutaneous and surgical myocardial revascularization^(3)^.

Aortic valve sclerosis has a 40% prevalence in octogenarian patients^(9)^, and initiates the process of calcific aortic valve stenosis, in which mineralization of the cusp has a pathophysiological mechanism similar to vascular calcification^(10)^. Calcific aortic valve stenosis is a predictor of cardiovascular risk in the elderly^(11)^. In opposition to control valves, calcified aortic valves express more alkaline phosphatase and matrix metalloproteinase-2^(12)^. Despite the calcified degeneration of the aortic valve being associated with atherosclerosis and its risk factors, studies that used statins to treat patients with aortic valve stenosis did not demonstrate decreased aortic valve stenosis progression ^(13)^.

## PATHOPHYSIOLOGICAL AND CLINICAL BACKGROUND OF VASCULAR CALCIFICATION

Epidemiologically, chronic renal failure (CRF), diabetes mellitus, and atherosclerosis are the clinical conditions that most contribute towards development of vascular calcification of medial and intimal layers of the vessel wall (Chart 1). In the real world, however, vascular calcification occurs indistinctly in these vascular layers, with different aspects due to variability, intensity, and coexistence of risk factors and disease in the population. In order to didactically discuss this, here we expose the contribution of a few comorbidities, risk factors, and respective clinical and laboratory changes that may imply in vascular calcification pathophysiology, despite the fact that often such conditions coexist and connect among themselves, such as the case of diabetes relative to atherosclerosis and CRF and vice-versa. Thus, the most frequent etiology of CRF is diabetic glomerulosclerosis, representing 50% of individuals with CRF. CRF is a clinical situation, in which secondary hyperparathyroidism is common, implying in increased intact parathormone, with consequent changes in plasma vitamin D3, calcium, phosphorus, and an increase of calcium-phosphorus product, primarily due to an increase in phosphorus^(8)^. Furthermore, patients with CRF have decreased levels of the vascular calcification inhibitor fetuin-A, (in which there is increased activity of osteoclasts, including bone fractures) and primary hyperparathyroidism due to a primary tumor of the parathyroid glands, can also promote vascular calcification.

**Chart 1:** Forms of vascular calcification, most frequent clinical settings, and risk factors in general population and in patients with chronic renal failure^(8)^

Lesion	Clinical manifestation	Possible risk factors in the general population	Possible risk factors in patients with CRF
Atherosclerosis: Plaques or circumferential lesions	Coronary artery disease, sudden death, peripheral vascular d isease, stenosis of the renal artery	Genetic predisposition, smoking, arterial hypertension, diabetes, dyslipidemia, inflammation	Traditional risk factors, besides disturbances of bone metabolism, products of advanced glycation (AGEs), oxidative stress, and inflammation
Calcification of the media	Increased pulse pressure, ischemia of multiple organs, aortic valve stenosis	Diabetes, aging	Diabetes, aging, disturbances of bone metabolism, AGEs, oxidative stress and inflammation
Uremic calcific arteriolopathy (calciphylaxis)	Ischemic skin lesions	Not observed	Disturbances of bone metabolism, especially hyperphosphatemia, obesity, female gender, and caucasian

CRF: chorinic renal failure.

Recently, the presence of regulatory proteins along with dedifferentiated osteoblast-like cells, originated from vascular smooth muscle cells (VSMCs) and called calcifying vascular cells (CVCs) was demonstrated ^(15)^. These cells are implicated in the synthesis/reabsorption of bone in atherosclerotic plaques, especially around calcification^(5)^. Therefore, it has been proposed that bone formation in the vessel is, in some aspects, similar to bone^(7)^. However, we know that in order to explain the paradox of osteoporosis/vascular calcification, a very common clinical situation in the geriatric population, in which vascular calcification prevails in opposition to skeletal bone formation, certainly the regulation of bone synthesis in the vessel and in the skeleton are different. This was studied *in vitro,* in which osteoblasts of the skeleton and CVCs (a population of vascular cells with osteoblastic characteristics) were cultured and showed opposing response (decrease and increase of bone secretion, respectively) after stimulation with oxidized LCL (LDLox)^(6)^ or after oxidative stress^(16)^.

Chronic inflammatory processes such as atherosclerosis may contribute to calcification. This is triggered from the response to injury caused by LDLox, which initiates the inflammatory process, amplified by the exposure of adhesion molecules, secretion of interleukins, CRP, and bone morphogenetic proteins (BMP) by endothelial cells and smooth muscle cells. Remarkably, this occurs in vascular topography with increased oscillatory shear stress, such as in vascular bifurcations. All these processes promote increased oxidative stress and decreased calcifying inhibitors, such as matrix Gla- protein (MGP) and osteopontin (OPN).

There are experimental evidence, implying that atherosclerotic inflammatory activity has an interrelationship with osteogenic modulation. When exposed to LDLox, endothelial cells express BMPs. Additionally, tumor necrosis factor-alpha (TNF-α) and interferon-gamma interferon (IFN-γ) stimulate the endothelium to express osteoprotegerin (OPG), which is also observed in osteoblasts and in smooth muscle cells when stimulated with interleukins (IL)^(17)^.

In patients with diabetes or hypertension, CRP and IL-6 correlate with serum levels of OPG^(17)^. In a recent study, high serum levels of IL-6 or reduced levels of IL-8 and IL-13 were independent predictors of coronary artery calcification (CAC)^(18)^. Another study demonstrated a complementary relationship between CAC, LDLox, and the monocyte chemotactic protein-1 (MCP-1)^(19)^. On the other hand, a meta-analysis failed to demonstrate a correlation among some inflammatory markers and CAC in patients with stable coronary artery disease^(18)^. Moreover, despite the potential anti-inflammatory effect of statins in patients with coronary artery disease (CAD)^(20)^, it is not clear if this benefit can decrease the progression of vascular calcification^(21)^.

Considering the potential cross-talk between inflammatory activity and the regulation of osteogenesis modulating proteins (OMPs), we can suppose that patients with acute myocardial infarction (AMI) can demonstrate a transient elevation in serum levels of these proteins as an acute phase response. In fact, one study recently demonstrated that patients with AMI had increased serum OPN, which peaked on the third day, reaching higher levels in comparison to healthy individuals^(22)^.

Reciprocally, higher CRP in patients with CRF, implying an exacerbated inflammatory state, stratify them as increased cardiovascular risk^(23)^, denoting another possible mechanism of pathophysiologic interaction. Furthermore, patients with a history of myocardial infarction and higher serum levels of phosphorus had a greater risk of cardiovascular events^(24)^. Finally, patients in the general community with upper quartile serum phosphorus levels and who had not suffered a myocardial infarction, also had an increased cardiovascular risk when compared to the lowest quartile^(25)^, even when adjusted for age and other risk factors, including CRP.

## DEDIFFERENTIATION OF VASCULAR SMOOTH CELLS INTO AN OSTEOCHONDROGENIC PHENOTYPE

**Figure 1:**
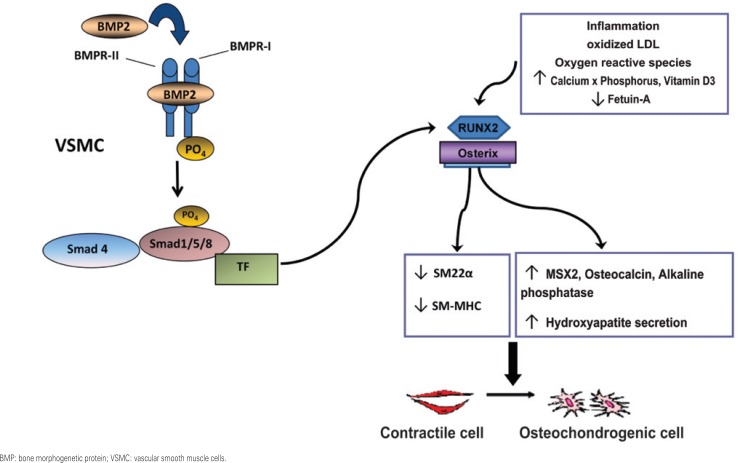
Dedifferentiation of vascular smooth cells into osteochondrogenic cells, resulting in vascular calcification. BMP2 binds to BMPRII receptor, creating a heterodimer with BMPRI, which activates intracellular signaling by Smad1/5/8 phosphorylation, then complexing with Smad4. In the nucleus, it leads to increased RUNX2 expression, along with other transcription factors. On the other hand, RUNX2 increases Osterix expression, another important calcifying modulator. In smooth muscle cells, this signal changes cellular phenotype, which is characterized by a decrease in contractile smooth muscle cell markers expression (SM22a and SM-MHC) and an increase in osteochondrogenic markers expression (alkaline phosphatase and osteocalcin), then finally promoting hydroxyapatite secretion. Other agonists of vascular calcification are shown on the right, such as inflammatory mediators, reactive oxygen species, oxidized LDL, phosphorus, vitamin D3, and decreased fetuin-A^(27,38)^

Calcification of the intimal and/or medial vascular layer (Chart 1) may also occur in the absence of an increased calcium-phosphorus product, orchestrated by BMPs, e.g. BMP2 and BMP4, and in presence of LDLox, such as in atherosclerosis. This process leads to differentiation of osteoblasts (CVC), whether from a smooth muscle cell, a mesenchymal cell, or vascular pericyte, with consequent increased expression of RUNX2, Osterix (Figure 1), determining an increment in alkaline phosphatase activity, osteocalcin production and bone matrix secretion^(26)^.

Corroborating the hypothesis of an active and organized process^(27)^, vascular calcification is initiated by the presence of vesicular structures in the matrix of ossified tissues and of atherosclerotic plaques^(26)^. These matrix vesicles are structures connected by membranes that detach from chondrocytes and osteoblasts, normally found in cartilage and bone, whose function is nucleation and growth of calcium crystals. Additionally, the existence of specific bone proteins in atherosclerotic plaques^(28)^, such as OPN, RUNX2 or Cbfa-1 (core-binding factor alpha-1, a specific marker of osteoblastic differentiation), OPG, BMP-2, MGP, alkaline phosphatase and receptor activator of nuclear factor kappa-B ligand (RANKL), implies *in situ* signaling mediators of bone formation and reabsorption in the vessel wall^(26)^.

VSMCs include a subpopulation of cells with different phenotypes. As the VSMC in culture initiate osteogenic differentiation (Figure 1), they lose the expression of smooth muscle specific markers^(27)^, indicating cellular dedifferentiation. A subpopulation of VSMCs, similar to osteoblasts, spontaneously express osteogenic proteins^(27)^ and produce a mineralized matrix in culture^(7)^. This subpopulation of cells, called CVC^(15)^, retains the capacity to differentiate into other mesenchymal lineages besides osteoblasts. Human VSMCs also express a receptor for vitamin D3 and the enzyme 25-hydroxyvitamin D3-1α-hydroxylase, which can be regulated when cultivated with parathormone (PTH) and estrogenic compounds^(29)^. Also, the dedifferentiation of these cells also depends on the sodium-dependent phosphate cotransporter, Pit-1^(30)^.

## OSTEOGENIC MODULATING PROTEINS

Osteogenic differentiation of VSMC has a pivotal role in progression of vascular calcification. VSMCs differentiation is continually modulated by oxidative stress, hormone and metabolic disturbance, and particularly, by osteogenic modulating proteins (PMOs). As we discuss in further detail below, PMOs can promote vascular calcification directly (RANKL and BMPs) or, ultimately, serve as constitutive biomarkers of vascular calcification (OPG and OPN). Additionally, besides the mechanistic aspects related to PMOs, there is also clinical interest. Serum levels of OPG, for example, have already been identified as a CAC biomarker in patients with stable coronary artery disease and as a prognostic marker in patients with AMI^(31)^.

*RANK-RANKL-OPG* is a pathway of interest and yet with undefined role in progression of vascular calcification in clinical settings. It implies the interaction between the receptor activator of nuclear factor kappa-B (RANK) with its ligand (RANKL) and the inhibitory function of OPG (as a decoy) on this interaction. OPG reduces the activity of the nuclear factor κ*B*, a transcription factor that regulates immune-mediated genes, and is important for inflammatory activity, innate immunity, and cellular differentiation^(32)^. RANKL is expressed in osteoblasts, smooth muscle cells, T lymphocytes, and stromal cells, while OPG is expressed in these cells besides the endothelium.

Regarding bone remodelling (reabsorption), activation of RANK by RANKL transforms preosteoclasts into their mature form^(33)^. This interaction is directly inhibited by OPG. However, the role of osteoclasts in vascular calcification and in bone reabsorption in the vessel is still undetermined.

**Figure 2:**
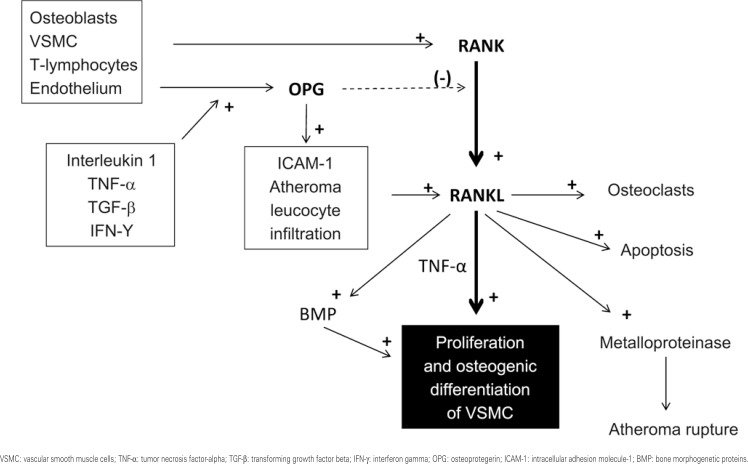
Signaling pathways in RANK-RANKL-OPG axis. Atherosclerotic inflammatory activity can promote smooth muscle cells osteochondrogenic differentiation

On the other hand, it is known that the *RANK-RANKL-OPG* axis participates in various stages of a complex atherosclerotic inflammatory cascade (Figure 2). RANKL is expressed in smooth muscle cells and in T lymphocytes, modulating dendritic cells maturation and inhibiting apoptosis..This activity may be inhibited by the action of OPG on RANKL. OPG, on the other hand, is stimulated by multiple inflammatory mediators, such as IL-1, TNF-α, TGF-β, and IFN-γ. These mediators increase endothelial OPG synthesis. Consequently, OPG stimulates adhesion molecules expression and leukocyte infiltration in the vessel wall. This process promotes the expression of RANKL, and the proliferation of smooth muscle cells^(31)^. Subsequently, RANKL directly stimulates osteogenic differentiation of these cells, or, indirectly promotes osteogenesis by increasing TNF-α secretion in monocytes^(34)^ or via BMPs^(35)^. The presence of RANKL in combination with OPG (particularly with a reduced OPG/RANKL ratio) increases metalloproteinase activity, which plays an important role in atherosclerotic plaque erosion and rupture^(36)^.

BMP2, which is part of the TGF-β superfamily, was originally identified as a regulator of cartilage and bone formation. This protein stimulates osteoblast differentiation^(35)^, and its presence in the atherosclerotic plaque suggests that arterial calcification is an actively regulated process, similar to bone formation^(7,37)^, as we further discussed in detail in (Figure 1)^(38)^.

There are at least 20 functionally and structurally related BMPs, most of them with an embryogenic and morphogenic function in various tissues and organs. Biologically active BMPs are usually homodimers containing a cysteine bond in their structure. The heterodimers of BMP2/BMP7 and BMP4/BMP7 may also exist *in vivo*. Heterodimers' activity may be regulated by a different or additional subtype of receptor and are more potent in stimulating bone synthesis and in inducing ventral mesoderm in comparison to homodimers. BMPs are extremely important proteins for cellular development and differentiation in the embryo and are phylogenetically preserved. BMP is ubiquitous (from insects to human beings) and its mutation/deletion or change in its receptor are lethal for embryo development^(26)^. It is known that increased expression of ossifying proteins occurs in mesenchymal cells or even in C2C12 myoblatic cells, which activate their osteochondrogenic gene programming after BMP2 stimulation for example^(39)^, to finally differentiate into osteoblasts, but not into smooth muscle cells, or adipocytes. In the vascular wall, the BMPs are possibly secreted by multiple cell lines, such as endothelial cells, smooth muscle cells, mesenchymal cells, pericytes, and inflammatory cells^(38)^.

## EXPERIMENTAL MODELS OF VASCULAR CALCIFICATION

Isolated hypervitaminosis D is a widely used model of *in vivo* vascular calcification, which has been studied for more than 40 years^(8)^. Various doses and forms of administration were described, although most researchers prefer to administer potentially toxic ammounts of vitamin D for a short time, resulting in acute hypercalcemia and hyperphosphatemia. A least toxic (with lower dose of vitamin D) chronic treatment also resulted in metastatic calcification and deterioration of renal function^(8)^. Broadly, administration of vitamin D results in calcification of the arterial wall and in a variety of other “arteriosclerosis” changes, such as collagen loss and rupture of elastic fibers^(4)^, finally causing increased aortic rigidity^(2)^.

Another model of vascular calcification described, uses topical application of CaCl_2_ 0.5M on aorta of rabbits^(8,33)^ or other mammals (rats and mice). The segment of artery submitted to CaCl_2_ becomes aneurismatic, with calcification of the media, inflammation, elastin mineralization and degradation, with disorganization of the extracellular matrix and increased matrix metalloproteinases^(7,8,33)^.

The *in vitro* model of VSMC calcification was described, by isolating CVCs, a subpopulation of bovine smooth muscle cell in culture, in which multicellular nodules appeared spontaneously^(33)^. Besides expressing OPN, osteonectin, and collagen-1, increased alkaline phosphatase activity and producing osteocalcin and hydroxyapatite, these cells were stimulated to further create calcification nodules especially when exposed to oxysterol 25-hydroxycholesterol, as well as to TGFβ-1, both present in atherosclerotic plaques. This model was then improved by incubation of these cells with a donor of inorganic phosphate, β-glycerophosphate 10mM, resulting in diffuse calcification after 14 days of culture^(15)^. Mineralization decreased with levamisole, an alkaline phosphatase unspecific inhibitor. Additionally, when 1.25(OH)-vitamin D3 was associated with β-glycerophosphate in VSMCs, calcification progressively increased with escalating concentrations, especially by decreased expression of an endogenous peptide similar to the parathyroid hormone (PTHrP) in these cells, which is an endogenous inhibitor of vascular calcification^(33)^. Additionally, the stimulatory effect of 1,25(OH)-vitamin D3 on alkaline phosphatase activity and OPN expression may contribute to promote vascular calcification. Pit-1 is a type III sodium-dependent phosphate cotransporter, which plays an important role in cellular mineralization, incubated with β-glycerophosphate^(30)^. We expanded this model, by demonstrating that BMP2, which is present in atherosclerotic plaques^(35,38,40)^, stimulates calcification of human coronary artery smooth muscle cells in culture by activating specific osteogenic signaling (Figure 1), implying in decreased smooth muscle markers expression and in increased osteochondrogenic proteins expression ^(37–40)^.

## FINAL REMARKS

Distinct therapeutic approaches of multiple pathophysiological pathways are necessary in order to decrease the progression of vascular calcification. Certainly, it is important to attenuate the impact and control cardiovascular risk factors^(9)^ such as dyslipidemia, diabetes mellitus, systemic arterial hypertension, renal failure, uremia and calcium-phosphorus metabolism imbalance. Some studies have demonstrated that statins, vitamin D and its analogues, and bisphosphonates seem to decrease the progression of the disease in specific patients^(8,13,21,26,33)^.

## CONCLUSIONS

Currently, vascular calcification is regulated by a complex pathophysiological mechanism, primarily triggered when there is an imbalance, in favor of osteogenic proteins and transcription factors synthesis, in detriment of bone reabsorption mediators. A better understanding of this pathophysiology is essential for exploring new therapeutic proposals and potential prognostic markers, in order to decrease the progression of vascular calcification.
